# Applications of Fusion Techniques in E-Commerce Environments: A Literature Review

**DOI:** 10.3390/s22113998

**Published:** 2022-05-25

**Authors:** Emmanouil Daskalakis, Konstantina Remoundou, Nikolaos Peppes, Theodoros Alexakis, Konstantinos Demestichas, Evgenia Adamopoulou, Efstathios Sykas

**Affiliations:** Institute of Communication and Computer Systems, National Technical University of Athens, 15773 Athens, Greece; edaskalakis@cn.ntua.gr (E.D.); kremoundou@cn.ntua.gr (K.R.); npeppes@cn.ntua.gr (N.P.); talexakis@cn.ntua.gr (T.A.); cdemest@cn.ntua.gr (K.D.); sykas@cn.ntua.gr (E.S.)

**Keywords:** data fusion, big data, machine learning, IoT, e-commerce

## Abstract

The extreme rise of the Internet of Things and the increasing access of people to web applications have led to the expanding use of diverse e-commerce solutions, which was even more obvious during the COVID-19 pandemic. Large amounts of heterogeneous data from multiple sources reside in e-commerce environments and are often characterized by data source inaccuracy and unreliability. In this regard, various fusion techniques can play a crucial role in addressing such challenges and are extensively used in numerous e-commerce applications. This paper’s goal is to conduct an academic literature review of prominent fusion-based solutions that can assist in tackling the everyday challenges the e-commerce environments face as well as in their needs to make more accurate and better business decisions. For categorizing the solutions, a novel 4-fold categorization approach is introduced including product-related, economy-related, business-related, and consumer-related solutions, followed by relevant subcategorizations, based on the wide variety of challenges faced by e-commerce. Results from the 65 fusion-related solutions included in the paper show a great variety of different fusion applications, focusing on the fusion of already existing models and algorithms as well as the existence of a large number of different machine learning techniques focusing on the same e-commerce-related challenge.

## 1. Introduction

Various definitions about data fusion can be found in the scientific literature [1]. According to White [2], data fusion is a process that encompasses the association, correlation, and combination of data and information from single or multiple sources in order to achieve refined position and identity estimations as well as in order to achieve completion and timely assessments of situations and threats, and their significance. Data fusion is characterized by continuous refinements of its estimations and assessments as well as by the evaluation of the need for additional sources, or the modification of the process itself, to achieve improved results.

Based on the above, it is obvious that fusion can assist in a lot of challenges faced in the everyday workflow of a company or business. More specifically, seeking the right information and knowledge in supporting human decision-making and other activities remains one of the main goals for all organizations and profit-seeking companies. Due to this need, the interest in information/data fusion techniques is increasing exponentially. For example, typical applications of these techniques revolve around the preprocessing step, the data modeling, as well as around problems concerning how to combine or fuse data from multiple sources in order to support decision making. Traditionally, focus has been given on fusing online sensor data [3,4], but more recent works also consider other sources as well, such as databases, simulations, ontologies, text documents, the web, and even humans. In addition, as the access and adoption of the internet are increasing at a rapid pace worldwide, the users of the internet and especially the number of buyers have also increased and keep increasing every year. In that light, over the last decade, e-commerce has become an essential part of people’s lives as well as of the global retail framework, with e-retail sales having grown more than 25% globally. The COVID-19 pandemic also played an important role in this increase due to the reduced ability of physical and face-to-face interaction and purchase of goods [5].

Under these circumstances, e-commerce ecosystems have been forced to evolve into a highly distributed critical infrastructure service, providing various kinds of different services such as shopping and payment, followed by delivery in physical or virtual form. Since e-commerce refers to the online transactions of selling goods and services on the internet, either in one transaction or through an ongoing transaction and because of the aforementioned reasons, companies have been forced to deal with handling heterogeneous big data, making the management of those data a challenge and making them seek for real-time, quick, and cost-effective solutions. In most cases, e-commerce firms deal with both structured and unstructured data. Structured data focuses on demographic data including name, age, gender, date of birth, address, and preferences, while unstructured data includes clicks, likes, links, tweets, voices, etc. [6]. More precisely, the types of data (e.g., orders, baskets, visits, users, referring links, keywords, catalogues browsing, social data), can be broadly classified into four categories; (a) transaction or business activity data, (b) click-stream data, (c) video data, and (d) voice data. To provide the reader with a better understanding of how fusion techniques provide solutions that can assist in addressing various issues related to e-commerce environments, this paper introduces a 4-fold main categorization of the analyzed solutions into product-related, economy-related, business-related, and consumer-related. Details about the main categorization as well as about the subcategorization of the solutions are provided in the following Section 3. To the best of the authors’ knowledge, such a categorization of fusion-related applications for e-commerce does not already exist. Furthermore, this paper also aims to facilitate similar future research works in the same area, where researchers can utilize the developed categorization or a similar version of it in order to organize their research findings.

The remainder of this paper is structured as follows: In the next section—Section 2—we take a dive into categorizations of the fusion techniques broadly used in the literature and the needs they assist on meeting through the algorithms they focus on as well as into popular methodologies utilized for fusion. Following that, in Section 3, a description of the methodology and the steps followed for this literature review are presented, together with details about the categorization of the analyzed solutions. In Section 4, the fusion-related applications in e-commerce are presented, based on the categorization already mentioned. Finally, in Section 5 we analyze the results of the current literature review, draw conclusions, and propose future research directions.

## 2. Data Fusion Techniques in Literature Categorization 

Data fusion is a rather broad term involving various fields. As such, the task of classifying fusion techniques is very challenging. Several classification criteria can be found in the literature [7,8], some indicative examples of which are provided in Table 1.

A non-exhaustive list of examples of fusion techniques is provided below, focusing on three main aspects related to data fusion, i.e., data association, state estimation, and decision fusion [12]. Data association refers to the process of assigning and calculating the weights that relate observations or tracks from one set to the observation/tracks of another set [13]. Some popular methodologies to address data association problems are: Nearest Neighbors (NN) [14], k-Nearest Neighbors (kNN) [15], Probabilistic Data Association (PDA) [16], Joint Probabilistic Data Association (JPDA) [17], Multiple Hypothesis Test (MHT) [18], Bayesian Networks, Markov Random Fields [19], etc. Popular methodologies for state estimation of a complex system include the: Maximum Likelihood estimation (ML) [20], Kalman filters [21], particle filters [22], covariance intersection and covariance union techniques [23], etc. The term Decision fusion mainly refers to the combination of different decisions deriving from different classifiers into a common decision regarding an activity that has occurred [24]. Some examples of decision fusion include Bayesian inference [25], Dempster Shafer Inference [26], abductive reasoning utilizing neural networks [27] or fuzzy logic [28], utilization of semantic features for decision making [29], etc. Further analysis of the aforementioned methodologies is beyond the scope of the current literature survey.

In addition to the above and in the context of a bigger data-oriented approach [30], fusion is a very useful tool when there is a need to analyze a vast variety of heterogeneous data, since it can be used for:

- The preprocessing of data: when used in the preprocessing phase, fusion increases the quality of raw data before they are applied in any data mining methods. This can be divided in two main subcategories: registration and re-identification. Registration on one hand means that the data refer to the same location in the environment over the same period of time. On the other hand, re-identification is a technique related to data registration and it consists of identifying data corresponding to the same object;
- Building models: knowledge coming from data in hand is usually represented by means of a particular data model that is extracted from a database. However, the set of alternative models considered in the literature that tackle the same problem is very large. In that case, data fusion methods can be applied in the process of model building and can be used in two ways: to define the model, meaning that a particular aggregation operator is used for combining a set of inputs to obtain a given output, as well as in order to combine several data models;
- Extract/mining information: a third use of information fusion is for extracting information to build summaries or a representation from the original data. This category can also include the dimensionality reduction methods.

## 3. Stages of the Literature Review and Categorization of the Analyzed Applications

**Figure 1:**
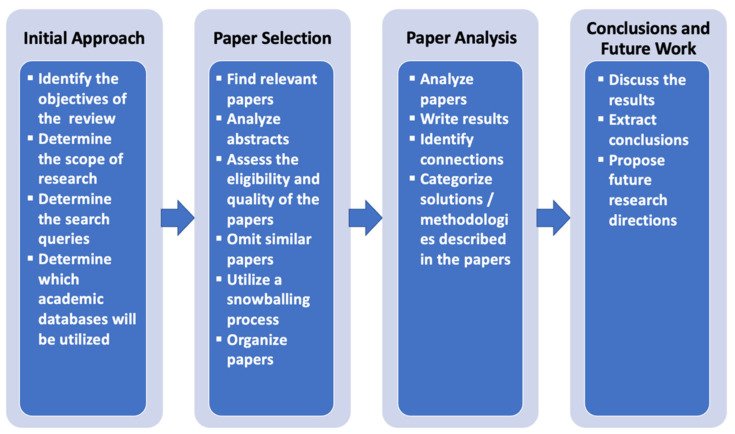
Stages of the present literature review.

The main steps followed in the context of this literature review are described in Figure 1.

As a first step, we identified the objectives of the review as well as the main scope and search queries relevant to our subject. Soon after that, we utilized the SCOPUS academic database for scientific publications as well as the Google Scholar search engine to find relevant papers. We reviewed 98 papers and analyzed 65 applications of fusion techniques in e-commerce environments. A total of 33 papers were omitted from our literature review, since they either had many similarities with other papers that were analyzed, or because the use of fusion techniques was not explicitly stated. We also utilized a snowballing process, where some of the research works that were cited in the analyzed papers were also analyzed. After analyzing these papers and identifying connections, we created a 4-fold categorization of the analyzed applications. More specifically, we classified the results in 4 main categories and 15 subcategories. Finally, in the final section of this paper we drew conclusions and proposed future research directions. 

The main search queries we utilized contained the following keywords or a combination of them: information fusion, data fusion, solution, algorithm, e-commerce, electronic commerce, retail, challenges, products, services, classification, fraud, detection, prediction, business intelligence, financial, economic, price, quality, recommendation, systems, marketing. In order for the reader to have a clearer view of the analyzed applications, the main categorization is comprised of the following four categories:

- Product-related;
- Economics-related;
- Business-related;
- Customer-related.

Many of the analyzed solutions do not focus on one specific challenge related to e-commerce but may provide a cumulative solution, addressing several different challenges at once. As a result, one citation might be relevant to several categories. However, our main criterion for the categorization was on which domain each application mostly focuses on. In other words, the existence of one application in one specific category does not exclude the possibility for this application to be relevant to other categories as well.

The 65 applications analyzed in the context of this paper can be found in the following section. The main categories, the respective subcategories as well as the citations for the applications analyzed in the context of this paper, can be found in Table 2 of Section 4.5.

## 4. Results

### 4.1. Product-Related

#### 4.1.1. Product Classification/Description

One of the main tasks identified in e-commerce platforms is the product classification task, for which the applications found can range from personalized search and recommendations to query understanding. A well-adjusted categorization of the products provides the customers with a better shopping experience. 

In that light, Zahavy et al. [31], suggested a multi-modal architecture for the classification of products in an e-commerce system, which utilizes a decision-level fusion approach. This approach leverages image and text classification results and fuses them into a multi-modal architecture that yields better performance compared to when image or text classification results are used separately. The authors also pointed out that feature level fusion methods require using the image signal for each product, while decision level fusion methods require using the image network selectively, making them more appealing. They also underlined that decision-level fusion performs better than feature-level fusion in practice. On the other hand, Verma et al. investigated the domain adaptability of state-of-the-art text and image modality-based architectures for the e-commerce product classification task, observing that the Bidirectional Encoder Representations from Transformers (BERT)-based textual classification models outperform the visual features-based models for this specific product classification task. However, incorporating both the modalities helped the authors leverage the complementary information present across the features, thereby enhancing the overall performance of the classification system by proposing a Deep Multi-Modal Multi-Level Fusion framework, which learns the joint representation using both the modalities simultaneously, where these representations are combined with the unimodal baselines in a probability-fusion strategy to boost the product classification system [32]. In addition, Zhao et al. [33] proposed a strategy based on deep flow truncation in order to create a new gradient truncation convolutional network by improving the multipath integration dense connection block and thus providing a more accurate way of identifying and classifying images of goods. In this paper, a fusion feature pyramid convolution network was constructed to define the feature graph of the DenseNet layers, which was then used in order to fuse the three layers feature maps through a Dual-Path Feature Fusion (DPFM) module, allowing the prediction layer to receive enough semantic and location data to accurately identify objects.

Finally, in the e-commerce product categorization solution proposed by Yu et al. [34], different classification models were fused based on simple voting, which refers to voting on the results according to multiple models, and weight voting method, which works by adding up the predicted probability values of multiple models and choosing the prediction with the highest probability value. Two types of models were used for single and multi-label prediction with the Fasttext [35], Text-Convolutional Neural Network (CNN) [36], Text-Recurrent Neural Network (RNN) [37], Very Deep Convolutional Neural Network (VDCNN) [38], and bi-directional long short-term memory (AbLSTM) [39] modules for the first model while for the multi-label prediction a hierarchical search tree and short path tree model were applied. The classification models were tested on an online dataset and were found to yield satisfactory results in terms of accuracy, recall, and F1-score metrics.

On the other hand, as far as product description methodologies are concerned, two common problems in e-commerce product description scenarios, where different modalities (e.g., text, image, videos) are utilized for product-based application tasks, are the modality-missing and modality-noise problems. One example of the first problem is when a seller uploads a description for a product while omitting a product image and/or title. One other example, this time for the modality-noise problem, is when the seller uploads a product image without the appropriate semantics and/or theme. Zhu et al. proposed a new methodology for multi-modal pretraining for e-commerce applications. This methodology encompasses three layers. In the modal-encoding layer, features from each modality are extracted, followed by a modal-task layer with diverse training tasks for each modality. Finally, in the modal interaction layer, the authors aggregated the information of the nodes and design a feature fusion model, where the initial image and text modalities were fused with their interactive features, leading to a better model performance. Experimental testing of the model on a real-world e-commerce dataset indicated important performance improvements over baseline and the state-of-the art methods [40].

#### 4.1.2. Customs Classification

Like product classification, customs classification is an essential procedure when importing cross-border goods traded by various companies and individuals, while its proper execution with high efficiency is still challenging considering the rapid growth of international trade.

In order to tackle that challenging task, Guo Li and Na Li [41] proposed a text-image dual CNN model based on text and image information to achieve automatic customs classification by designing a novel method to fuse the two CNN sub-models and improve the classification performance. To verify its efficiency and demonstrate the implementation of the model, they conducted a case study followed by training of the sub-models, in which they employ adaptive weights to combine the prediction results. On the other hand, Bilgehan Turhan et al. [42], in order to make a root extraction of words, proposed a morphological parsing system in the textual phrases. The authors later proceeded with finding the best matching and harmonized definitions within the system, which resulted in triggering a quantized local-based feature visual search. Finally, results on both visual and textual analysis are fused at the score level and the result of that fusion showed a significant increase in the accuracy of the results retrieved from the topic modeling module.

#### 4.1.3. Goods Information Inspection

Goods information inspection is a common challenge faced in the e-commerce environment since the maintenance of goods information and the malicious behaviors of some traders sometimes induce mismatches between the products or services and the corresponding information, causing false delivery and a bad customer experience. Within this scope, Liu et al. [43] proposed a model fusion method for semantic consistency inspection of information on both an online retailing platform and a logistics platform in order to ensure the successful and reliable delivery, by scanning the information on these platforms and analyzing the semantic consistency based on the text features. The paper explains that in order to guarantee the quality and reliability of inspection, this method leverages the complimentary effect of multiple learning-based features as well as the hand-crafted features created based on domain knowledge, resulting in a very high accuracy and fitting measures. On the other hand, work featured in Ref. [44] presents data fusion as a final comprehensive evaluation of the products’ results, based on three aspects: store reputation, costumer reviews, and a so-called product hot index. For this purpose, Pang L. et al. described the frameworks for multidimension synthetic evaluation that consist of four layers of the data gathering using the crawler tool to retrieve the information from the store, the data processing layer applied in numerical and textual information, the multi-dimensional information fusion layer, which describes the store and its products from different dimensions, and a single-dimensional indicator calculation layer using the Principal Component Analysis, which helps determine the key factors that affect the store reputation and product hot index.

#### 4.1.4. Goods Demand Forecasting

Product demand forecasting is an important step of the e-commerce supply chain and commodity inventory management that affects the company’s replenishment strategy and inventory cost reduction. The use of fusion techniques, when applied in this task, can improve the accuracy of forecasting results by studying the factors and combining these results. In that light, the paper by Cai et al. [45] proposed a novel methodology for commodity demand forecasting in e-commerce, which considers the long-distance dependence along with the short-distance dependence in sequential data while addressing the problem where the recurrent neural network pays attention to the long-distance dependence in sequential data only. The authors explain that the connection between two features is weak when there is a significant distance between them, due to the short distance dependence also resulting in less retained information. They also proposed a strategy which effectively gets the deep spatial relations among multimodal data by fusing each column’s features across spatial dimensions. Furthermore, Shi et al. also developed and proposed a framework [46] by incorporating a fusion network with multiple patterns and the meta-learning paradigm. In order to adjust the initialization of model parameter in the meta-learning paradigm, the segment relations are further distilled. The results on this paper regarding the comparison with the baseline method showed that the proposed framework is more effective in both offline and online scenarios. Finally, in Ref. [47], Zhang and Dong proposed a buyer prediction ensemble model based on the votestacking fusion method, which applies the subtime under sampling to process the unbalanced historical behavior data of buyers as a first step while the three individual models, namely DeepCatboost, DeepGBM, and DABiGRU, are constructed consecutively. Then, the vote-stacking fusion method is used to fuse the prediction results of the three individual models and obtain the final prediction results. The experimental results showed that the proposed model is superior to the reference models.

#### 4.1.5. Shipping and Route Optimization

Timely delivery of products is a very important, albeit challenging, part of the successful execution of an order in e-commerce. Proper management of the shipping procedures can result in important cost reduction as well as in an increased level of customer satisfaction. Those needs have led the e-commerce environment to reach for solutions that will assist on the timely and correct shipping of their goods.

Kandula et al. presented a framework for decision support, which has the goal of both increasing the delivery success rates and decreasing the delivery costs. This solution predicted the appropriate delivery time periods for successful deliveries. For this, machine learning models were utilized for the prediction models (i.e., Random Forests-RF, Extreme Gradient Boost-XGBoost, LogitBoost, Artificial Neural Networks-ANNs, and Decision Trees). Feature-level fusion was also utilized for the datasets that serve as inputs to the prediction models. Initially, predictions were created for the delivery success of each order in the delivery shift. These predictions were then utilized as inputs for generating optimized delivery schedules. Test evaluation of this framework showcased a reduction of 10.2% in the delivery costs as compared to the current delivery practice [48]. In addition, improper route selection, which is often noticed in cross-border e-commerce logistics distribution, results in reduced efficiency. Aiming to tackle this problem, Quan proposed an optimization technique that utilizes information fusion and the improved genetic algorithm. More specifically, information fusion was implemented to acquire the time window cost of logistics distribution in e-commerce as well as to detect safety hazards, optimize repair processing, select the optimal distribution branch, conduct weight bearing, and optimize the selection steps of the optimal e-commerce logistics distribution. Experimental results of the proposed solution showed increased transportation efficiency and better performance compared to the traditional route allocation methods [49]. Given the various classes of supplies and the specific traffic characteristics within a city, each road enables the delivery of each class of supplies with a specific probability, Yang and Wu proposed a logistics path optimization model for e-commerce, where they utilize feature fusion and a hybrid genetic algorithm. The main aim of the authors is to choose finite locations on a map as the centers of supplies, in order to maximize the number of locations that can be covered in an effective manner by the paths followed by the available vehicles. Results from testing the aforementioned novel approach indicated better performance than other contemporary methodologies [50]. Additionally, last-mile services offered in e-commerce frequently include delivery in indoor environments. While existing navigation systems (e.g., GPS) offer satisfactory localization accuracy in outdoor environments, they often struggle in indoor environments, not being able to provide satisfactory localization. Wang et al. presented a novel low-cost fusion-based localization algorithm for indoor environments that can run on low-cost Android operating smartphones and requires low battery consumption. For the navigation, data fusion is applied on data deriving from an IMU sensor as well as from an Oriented FAST and Rotated BRIEF Visual Simultaneous Localization and Mapping (ORB-SLAM) algorithm. The solution can be used for autonomous last-mile delivery using distribution robots. Experimental results indicated a satisfactory error value, which was limited within five centimeters in indoor navigation, while also resulting in battery consumption savings of about 56% [51].

At the other end of the spectrum, maritime transportation systems are also an important part of contemporary e-commerce operations. The growing demand for freight movement combined with stricter environmental regulations underline the importance of increasing the maritime efficiency. Towards this direction, Sugrue and Adriaens showcased a fusion-based methodology for Automatic Identification Systems (AISs) and navigation lock data. The authors also proposed a specific metric, called Maritime Transport efficiency (MTE), which is derived from the fusion of AISs and navigation lock data, incorporating the travel time and the vessel payload. They also presented a linear model for vessel capacity estimation as well as travel time statistics for bulk carriers. The efficiency estimation can be particularly useful for operations managers and can support near real-time decisions regarding fleet deployment and risk transfer mechanisms [52]. Another solution for maritime efficiency optimization was proposed by Spandonidis et al. [53]. In this paper the authors presented a platform that has the main goal of continuously gathering critical information from various ship’s inputs, processing data, and transmitting them wirelessly as well as analyzing the measurements to support the decision-making of shipping companies. The platform uses a hybrid data fusion pipeline as well as RNNs and, more specifically, Long Short-Term Memory (LSTM). Experimental testing of the platform for fuel oil prediction indicated that it could accurately predict the consumption, while keeping the error below 1% and the standard deviation of the error below 2%.

#### 4.1.6. Supply Chain Management

Supply chain management can be defined as the link which connects all the elements of the manufacturing and supply process. It includes processes from the entire value chain, addressing materials as well as the supply management from as early as the extraction of materials until the end of its useful life [54]. Wei and Wang [55] demonstrated a novel model for integrated supply chain management in cross-border e-commerce operations, which utilizes fuzzy C-means clustering. The authors created a sampling model of reliability characteristic information and conducted panel data fusion based on the results of regression analysis of risk sample data. Empirical analysis of this model indicated improved integration and management of the supply chain of cross-border e-commerce services. In addition, Supply Chain Collaboration (SCC). in which different market actors work jointly to execute supply chain operations, is of particular importance for e-commerce, where most organizations prefer to cooperate with reliable and sustainable companies. Ali et al. presented a fusion-based SCC solution [56], which utilized Support Vector Machine (SVM) and kNN algorithms. The authors also conceptualized a framework for effectively tackling disruption risks. In the context of this framework, relations were detected among samples, performance assessments, and the customer feedback. With the help of this framework, users can detect how and where data analytics can be integrated in SCC, relying on offline decision-making only. Experimental testing of the proposed approach showcased better accuracy as compared to other contemporary solutions for SCC.

Along with the cross-border supply management, efficient management of the food supply chain is also of high importance in e-commerce applications. Pang et al. proposed a value-oriented business and technology joint design framework for the food supply chain, where Internet of Things (IoT) and information fusion technologies are utilized. At first, the authors focused on the assessment and evaluation of the added value created by various factors, including shelf-life prediction, sales premium, assurance cost reduction, etc. Soon after that, they systematically created and implemented IoT sensor portfolios, taking into consideration causes of food spoilage, as well as energy and delivery costs. Finally, a fusion architecture was proposed by means of mapping the available data processing on a cooperative cloud. A prototype system was also implemented, confirming the feasibility and effectiveness of the proposed framework [57]. Another solution relevant to the food supply chain management that focuses on agricultural supply chain was presented by Sun and Shu [58]. Firstly, the authors demonstrated research on the perception data fusion in the agricultural product chain focusing on IoT technologies. Soon after that, they presented a sensory data fusion model for the supply of agricultural products. Experimental testing of the model indicated the benefits it provides on several aspects, including improvement of the supply efficiency, the improvement of logistics efficiency, tackling the problem of unknown source of agricultural products, reduction of health products, as well as reduction of the prices of agricultural products.

Finally, Ajitha et al. [59] demonstrated an application which ensures both safe purchase and safe delivery of the products by including three different modules: the Authentication Module, the Ordering and Scheduling Module, and the Tracking Module. In addition to the modules, the software proposed includes a k-mean clustering algorithm in order to find the shortest route available to the customer while the user is also able to track the delivery of the purchase with no delay. It can also classify the users as spammers and legitimate or not by creating a collection of YouTube users that is used to classify content, individual, and social attributes that help to characterize each class of users. The proposed hybrid research is applicable in the context of data fusion based on Dempster Shafer’s (D-S)—conceptual method and then on Adaptive neuro-fuzzy inference system (ANFIS).

### 4.2. Economic-Related

#### 4.2.1. Financial and Credit Risk Prediction

Organizations focusing on e-commerce, as any other cost-profit organization for that matter, have to deal with financial challenges due to high distress, incomplete financial information, and constrained capital. In that light, those kinds of challenges adversely influence the health and sustainability of the development of the companies, especially in the tough circumstances of financial crisis and the pandemic. To address this problem, there is a need for solutions specialized to optimize the financing circumstances. For that purpose, the solution proposed by Zhang et al. [60], in order to improve the prediction of credit risk, included a multi-modal deep learning methodology, having a two-component approach. In the one approach described, the authors suggested the fusion of the multi-source heterogeneous data by applying the multi-modal learning strategy (e.g., the static enterprise demographic data and the dynamic financing behavior data are fused). The other one used the concatenated vectors derived from data fusion as the input of the feed forward neural network (FNN) to predict the credit risk of Small and Medium-sized Enterprises (SMEs). The results showed that the fusion of the two different sources of data is superior to the existing studies on credit risk prediction of SMEs. In addition, as the consumer credit is at a relatively early stage in China and in order to quickly occupy market share and maintain an advantageous position, e-commerce companies have increased the annual growth rate of the total amount of credit and have lowered the conditions of credit through e-commerce. Aiming at the problem of e-commerce consumer credit default analysis, Hou et al. [61] proposed a Fusion Enhanced Cascade Model (FECM). This model learns feature data of credit data by fusing multi-granularity modules and incorporates RF and Gradient-Boosted Decision Trees (GBDT) trade-off variance and bias methods. The paper compared FECM and gcForest on multiple data sets, to prove the applicability of FECM in the field of e-commerce credit default prediction. The research results of this paper are helpful to the risk control of financial development, and to construct a relatively stable financial space for promoting the construction and development of e-commerce.

Finally, in order to have a better understanding of the big amount of economic data analyzed from various cross-disciplines (e.g., cloud computing, artificial intelligence and machine learning, macroeconomic forecasting, and microeconomic analysis) Liang et al. in [62] researched economic and security big data collection while focusing on multi-source heterogeneous data fusion algorithms and cleaning techniques in order to create a style suitable for data analysis of economic security. The authors also proceeded with the construction of different big data computing frameworks, real-time risk early-warning analysis algorithms for the real-time and delay needs of big data early-warning analysis of economic security, and deeply explored the relationship between different industries and regional economies.

#### 4.2.2. Price Prediction

Customers’ needs and budget play an important role when deciding to buy a product or a service, so the price of a product is very crucial to their market share. In that light, decision makers of a business—including e-commerce businesses—need to make proper price prediction for their products in order to achieve a good profit of the sales and have a healthy competitive strategy. For that purpose, the authors in Ref. [63] proposed a fusion model of machine learning techniques and business intelligence in order to apply an effective and profitable pricing mechanism of products. The machine learning algorithms, e.g., Multiclass RF, Multiclass Logistic Regression-LR, and Multiclass one-vs-all have been fused and applied for product price prediction and the result of this approach has led to a win-win situation for the customer as well as for the business by achieving a prediction of the product demand based on transactions and reviews of products, and the customers’ buying behavior to categorize them based on the amount spent. According to Mahoto et al., their model helps the customer purchase the desired product at an affordable price while the business accomplishes its goals by selling out the maximum number of products at a certain time and keeping its profit stable. On the other hand, the authors in [64] showcased a methodology for predicting auction end prices, which has the main aim of maximizing the profit of an e-commerce online auction platform. This proposed framework utilizes a fusion algorithm that leads to a more effective outcome and manages to overcome the shortcomings of the simple multiple LR algorithm. Finally, when cross-border e-commerce is conducted, the formulation of a proper pricing strategy that will help maximize the corporate profits is of high importance for e-commerce operators. Towards this direction, Guo proposed a methodology for automatically formulating an optimized pricing strategy for cross-border e-commerce. In this research work, the author considered various factors affecting the evaluations for products of customers from different countries and then used them to conclude a marketing share prediction. In the context of this solution, CNN-based image feature extraction took place and different attention mechanism strategies were developed to select image features related to the users’ evaluations on products. Then, out of all the influencing factors, the author focused on the three most important (i.e., the cross-elasticity coefficient, the tax difference, and the 3rd party platform usage fees) and performed a simulation study, resulting in the most suitable commodity pricing strategies for diverse scenarios [65].

#### 4.2.3. Financial and Credit Fraud Detection

Fraudulent transactions comprise one of the most crucial threats to e-commerce. They can be defined as transactions where the account and/or payment details of an individual are used without permission. These transactions can lead to financial loss, loss of property, as well as loss of information for the users. The research work conducted by Shinde et al. had the main goal of creating a model for fraudulent transaction prediction. The authors firstly benchmarked different machine learning models for fraud detection in terms of various metrics (e.g., accuracy, F1-score, Precision, Recall). More specifically, metrics were acquired for the following models: RF, XGBoost, LR, Gaussian Naïve Bayes (Gaussian NB), and Stochastic Gradient Descent (SGD). The authors then used a fusion methodology, where only the models with metrics above certain thresholds were utilized in order to maximize the performance. Experimental testing of the methodology showcased high prediction ability of fraudulent transactions with scores above 99% for all the aforementioned metrics [66]. Li et al. showcased an attention-based Heterogeneous Multi-view Graph Neural Network (aHMGNN) solution [67] which can be used in many applications, including a fraud detection system for e-commerce. The described solution modeled a more complex multi-view network where diverse node and edge types co-exist. Two stages were included regarding node embeddings learning and multi-node and edges representation fusion. Experimental offline and online testing on various datasets indicated high scalability, effectiveness, and efficiency in fraud transaction detection and other applications. In addition, Liu et al. [68] proposed a solution for financial fraud detection, utilizing e-commerce big data. Initially, behavior features are extracted with encoders. Soon after that, a neural network methodology was adopted, and feature fusion was executed by means of weighted correlation methods, thus improving the feature classification procedure. Then, financial fraud was detected by utilizing sparse reconstruction errors. Experimental testing of the proposed solution indicated the high capability of the methodology in learning essential characteristics of data as well as increased detection rate as compared to other contemporary fraud detection algorithms.

Fraudulent transactions in e-commerce are often coordinated by organized fraudsters. Under that perspective, Marchal and Szyller [69] proposed a novel methodology for the identification of groups of fraudulent orders, which are related to organized fraud. This methodology utilizes agglomerative clustering and sampling in order to detect organized fraud by analyzing large numbers of transactions in a short amount of time. In the context of this methodology, cluster fusion is utilized as well. Experimental testing was conducted on a large European online apparel retailer, where the methodology was able to analyze more than 100,000 orders in a few hours, grouping a substantial part of fraudulent orders together, while raising false alarms for only 0.1% of the orders. The solution was found to outperform other existing clustering techniques.

Finally, two popular fraud detection techniques relevant to e-commerce include shill biding detection and card fraud detection. The term shill biding refers to the cases where sellers introduce fake bidders in auctions, aiming to increase the final price. Abidi et al. proposed a fusion-based solution for detecting this kind of fraud based on SVM, ANNs, and fuzzy logic. The solution was comprised of three different modules. In the first one, an SVM and an ANN algorithm were trained on the same dataset to predict fraud. The prediction of each algorithm was used as an input to the fuzzy fusion model and the decision on whether there is a fraud or not is taken. Experimental results of the methodology indicated a high accuracy of 99.63% in shill biding detection, outperforming other state-of-the-art techniques [70]. Card fraud detection is also of crucial importance nowadays, as more and more users are using credit cards for their e-commerce transactions. In Ref. [71], Darwish proposed a credit card fraud detection solution from imbalanced datasets, which is based on the semantic fusion of K-means and Artificial Bee Colony (ABC) algorithms. This fusion contributes to the improvement of the classification accuracy and leads to faster detection convergence. With this fusion, different variables are associated by means of clustering levels with their meanings for consumers. Experimental testing showcased improved classification accuracy as compared to traditional card fraud detection methodologies.

### 4.3. Business-Related

#### 4.3.1. Business Intelligence and Decision Support

The term “Business Intelligence” refers to both a process and a product according to Vedder et al. As far as the process is concerned, it contains methodologies utilized by organizations to create useful information/intelligence to “survive and thrive in the global economy”. Regarding the product, it is the information which enables a corporation to successfully predict the “competitors, suppliers, customers, technologies, acquisitions, markets, products and services, and the general business environment” with a certain degree of certainty [72].

With the rapid development of Information and Communication (ICT) technologies, Business intelligence and Analytics (BI&A) are applied in more fields, with e-commerce being no exception. Emerging application scenarios encompassing heterogeneous data from various sources are highlighting the need for data fusion. Taking this need into consideration, Li et al. proposed a novel BI&A framework which included fusion at two levels, i.e., data level information level and knowledge level. This framework considered aspects regarding objects, methods, and humans. More specifically regarding the human aspects, the preferences of decision-makers, the experts’ opinions as well as social and cultural environment factors were taken into consideration. The authors presented economy, finance, and management as some of the potential areas where the proposed framework can be applied [73]. The so-called GVUCI model, which was an environmental adaptability model, was set as the information fusion model base and knowledge base as well. The D-S evidence theory model was selected as the fusion algorithm, and the decision support model of e-commerce ecosystem was constructed based on information fusion. This model could help enterprises to recognize the situation and forecast the potential changes. It could guide enterprises to make reasonable development strategy and had a positive theoretical and practical significance for e-commerce enterprises to obtain favorable competitive positions. The model in this study was the application of basic principles and algorithms of information fusion technology in the e-commerce ecosystem. However, the current theory of the e-commerce ecosystem is still not mature, and the system itself also has many uncertain factors and characteristics that also need further analysis and research [74]. Another human-centered framework was proposed by Huang et al., which was based on several consumer contexts to discover and generate business intelligence. As an initial step, consumer activity logs from the real world and the cyber world were collected. Soon after that, data analysis was conducted, making use of mining algorithms and knowledge-information-data (KID) fusion. This framework also utilizes behavior and computational psychological models and encompasses an open platform, which supports third-party contributions as well as the evolution of the human models used. The framework was also tested on a dataset regarding the online purchasing preferences of 800,000 users from an e-commerce website during the period 2006–2012. The results of the fusion of several algorithms (i.e., LR, NN, DT, NB, SVM, KNN) were found to yield 0.6–3.4% accuracy improvements as compared to the accuracy of the individual algorithms [75]. On the other hand, acquiring a better understanding of users’ needs and preferences is of vital importance for e-commerce. However, the acquisition and processing of such data and the extraction of useful information can be rather challenging tasks. Towards this direction, Sato et al. presented a fusion-based mining and analysis methodology. Firstly, the authors described a mining approach for acquiring data for users from real world, social world, and cyber world sources. In order to improve the mining engine, a three-layered procedure was proposed, utilizing fusion techniques and the Recency, Frequency, and Monetary Value (RFM) model. Finally, the authors presented a case study, where the aforementioned methodology is applied in a company with the goal of expanding its business interests [76].

Additionally, the enormous amount of data on users’ location information, has led to the challenge of mining useful information to support the decision services on mobile e-commerce. In order to improve the location information value coming from multiple users and promote the development of e-commerce push service, Xiaoyan et al. [77] proposed an algorithm using a fusion method on multisource information based on Monte Carlo. Under this light and in order for the sampling distribution to be as close to the real one as possible, the proposed algorithm used Unscented Kalman filter in order to build importance. For the processed data, an adaptive algorithm was used to avoid resampling, while to avoid the particle degeneracy phenomenon, the Metropolis–Hastings (MH) sampling algorithm was also used. Finally, Zhang et al., in their article [78], studied the big data decision intelligent perception system through a fusion method that combined the data fusion algorithm method, the data fusion network model, the quaternion method, the big data decision intelligent perception system framework design experiment, and the Cross-border e-commerce (CBEC) user experiment. Finally, in order to facilitate proper decision making regarding sustainable partners, Aslani et al. proposed a novel framework that combined information fusion and grey multi-criteria decision-making (MCDM). A shortlist of the best suppliers was created with two different methodologies. The first methodology combined grey best-worst method (BWM) and grey weighted aggregated sum product assessment (WASPAS). The second methodology combined grey BWM and grey technique for order of preference by similarity to ideal solution (TOPSIS). The rankings created by each of the above methodologies were integrated and the experts’ opinions were also inserted in the information fusion framework, thus producing a unified ranking of the suppliers taking into consideration several social and environmental aspects. During experimental use, the proposed framework was found to be very effective in sustainable partners selection in various industrial settings [79].

#### 4.3.2. Information Quality Assessment

Decision making’s success in a complex environment depends on being aware of and compensating for insufficient information quality at each step of information exchange. Good quality of input information does not always guarantee sufficient quality of the system output, but it is very important when raw data enter the system, and when information is transferred between automatic processes, between humans, and between automatic processes and humans. For those reasons, the paper described in Ref. [4] proposed a framework assessing the data quality based on user-defined strategies where the available data at the end of the procedure was sent as output. In this case, the integration and adaptation of concepts that are represented in different formats by different sources, was dealt with data fusion. The strategies mentioned by the authors include the elimination of insufficient quality information, the incorporation of information into models, data and information modifications by quality compensation, and a combination of the above approaches. On the other hand, Nahari et al. tried to tackle imprecisions, redundancy, and homogeneity of data as well as conflict derived from different data sources. In their paper [80] they proposed a fusion approach based on the similarity between two entities, which were passed to the so-called Linked Data Quality Assessment module, which was also described in the paper. In this module, similar entities were fused and then processed together to decrease conflicts and improve data quality.

In addition, one more problem faced when dealing with many data, especially personal data, is the duplicates or the similar entries about people. In order to tackle the issue of duplicate person instances, the authors in Ref. [81] proposed a machine and deep learning-based method, which is also reffered to as person fusion. In this approach, a comparative analysis was followed between different machine learning and deep learning methods for person instance verification with the use of two datasets, one balanced and one imbalanced, as input. Finally, after the preprocessing of the datasets, NN and LR were used as benchmark for both datasets, while for only the imbalanced one, RF and penalized Support Vector Classifier (SVC) were evaluated.

#### 4.3.3. Recommendation Systems

Recommendation systems are of high importance for e-commerce operators and can support and facilitate decision-making regarding various customer and business-related aspects. Guo et al. also proposed a fusion-based recommendation system for e-commerce based on location information and online behavior of customers in their smartphones or tablets. In this research work, evidence was extracted, and a radial basis function (RBF) neural network was utilized to calculate weights. Then a D-S methodology was used to fuse information and the power spectrum estimation to result in improved product recommendations. The efficiency of the proposed system was tested in a case study of an online dress shop. The results indicated that the performance was better than traditional recommendation methods regarding the recall and coverage rate and the accuracy [82]. User reviews are frequently used in collaborative recommendation systems. Khan and Mahalakshmi proposed a fusion-based aspect-mining recommendation system, utilizing both implicit and explicit reviews. Firstly, the system mined sentiments regarding different aspects from user reviews. These sentiments were then transformed to multi-dimensional ratings. The ratings were then fused with demographic and user profile data in order to provide high quality recommendations. Experimental results of the proposed solution indicate lower Root Mean Square Error (RMSE), Mean Attribute Error (MAE), and Mean Relative Error (MRE) than other contemporary collaborative filtering recommendation systems [83]. On the other hand, Lin et al. [84] proposed a Feature Fusion Deep Neural Network (FFDNN) methodology, aiming to face the problem of user-item matrix sparsity, which is commonly found in recommendation algorithms as well as to increase the overall recommendation accuracy of such algorithms. This methodology utilized the user scorings for items as well as text descriptions regarding products and user information. The eigenvectors of products and customers were constructed utilizing information fusion matrix decomposition, thus significantly reducing the aforementioned sparsity problem as well as the cold start problem regarding users and items. Experimental results indicated that the proposed solution outperformed traditional recommendation algorithms in terms of Root Mean Square Error and accuracy.

On the other hand, due to the different consumption behaviors and habits of the consumers in the different regions of a city, some information on the behaviors might get lost or be ignored, leading to false recommendations. In that light, Wang et al. [85] proposed a feature fusion personalized recommendation algorithm based on collaborative filtering combined with dense feature data and sparse feature data. This algorithm focused on learning the characteristics of users in a specific region and the characteristics of some sparse users while it also learned the time period of ordering, thus mitigating the model and personalizing the recommendation of the item the consumer may buy. Additionally, in order to capture the consumers’ interest indicators coming from multiple sources, due to the many emerging e-commerce websites and the consumers’ limited cognition as well as in order to identify their needs, Zhu [86] proposed a model based on the interest modeling method as well as on the limited interest indicators and the differences on attenuation from the user shopping process perspective. In this case, a data fusion strategy was used in the e-commerce service recommendation, based on three networks in order to meet the requirements and increase the impact of location from e-commerce mobile real-time recommendation. To address the same problem, Moreita et al. [87] proposed a multi-modal information architecture leveraged from textual, image, and tabular data, actually consisting of two architectures, Transformer-XL and XLNet. The information in this case was leveraged by exploring different kinds of data mined, like tables of user interaction events and unstructured data like descriptions of product and images, highlighting how the product popularity can affect the recommendation accuracy. Additionally, Li et al. in their attempt to fill the gap created by the cold-start problem when recommending a product to new users, developed through their study the Consensus Interest Prediction Model (CIPM) [88] in order to predict new-users satisfaction for the product that has been recommended. This was achieved by using the homogeneity in social commerce websites to improve the robustness of the prediction and deep extraction of user information. Five types of homogeneity were extracted for that purpose in order to use direct linear and nonlinear fusion and indirect fusion of multiple homogeneity indices in the design of the multidimensional homogeneity prediction models, which was then nominated for a given new recommendation. Finally, to estimate the similarity between the models, the prediction errors were calculated and the model with the highest consensus was selected through a weight voting algorithm.

#### 4.3.4. Marketing Optimization

According to Ref. [89], e-commerce marketing “is any marketing effort you do to promote your online store and generate sales. It applies both to getting new customers (customer acquisition) and making old ones shop again (customer retention).” Based on this definition, a lot of challenges emerge, which need to be taken into consideration for the e-commerce environment to have a smooth and healthy profitable plan. Liu’s research on the existing marketing methods [90] resulted in proposing a precision marketing analysis, based on optimizing the existing curling neural network analysis applied until now. The fusion applied into the network emphasized the better performance achieved as compared to the performance through the postprocessing of classification scores. The CNN model, LSTM model, LSTM attention model, and CNN and LSTM attention model were compared, with the performance of the CNN and LSTM attention model and LSTM attention model achieving the highest overall accuracy of testing and training. The recommendation results provided by marketing methodologies based on a single model sometimes provide unsatisfactory results regarding the advertising conversion rate prediction. Aiming to achieve better recommendation results, Zhao et al. presented a marketing solution for e-commerce, which was based on multimodel fusion—Artificial Intelligence (AI) fusion and Big Data. The authors began by introducing a Big Data technology and an analysis of the characteristics of the Real Time Bidding (RTB) advertising model. Then the solution utilized multitask learning and fusion technologies. Soon after that, the importance of specific advertising words for online marketing was calculated based on the Term Frequency-Inverse Document Frequency (TF-IDF) technology. The marketing effect is classified based on multitask fusion and XGBoost methodologies. Experimental results indicated that the prediction results of the proposed model outperformed methodologies based on a single model and also resulted in increased online sales [91].

On the other hand, Liao and Tsai demonstrated a novel algorithm for Business-to-Customer (B2C) marketing based on a Least Square Support Vector Machine (LS-SVM) methodology. The proposed model encompassed influencing factors of the consumers purchasing behavior. After developing an initial version of the model, the authors analyzed the data from a specially designed questionnaire for e-commerce consumers to optimize the model. The extraction of marketing strategies was based on five main pillars, i.e., channel and brand building, the positioning of the product, the website design, as well as the pricing strategy. The multi-model fusion was implemented with multi-observation samples, representing the specific mode of online B2C online marketing. The algorithm began with an assumption of the label of the sample set and then transformed it into the constraint condition of the optimization problem. It then calculated the multi-model fusion marketing error. Then a multi-model fusion-based stacking integrated methodology was implemented to result in a multi machine learning algorithm which was embedded in the B2C prediction model. Experimental testing of this methodology indicated high robustness and better performance than other B2C algorithms utilizing multi-model fusion [92]. Following the same idea, Zhang et al. introduced a methodology for precise marketing data mining in e-commerce platforms, which utilized information fusion and distribution similarity analysis. The authors firstly described how the optimization of the original system hardware framework took place. This optimization facilitated the data collection and made sure that the proposed software development could be realized. Soon after that the customer trajectory model was described, which analyzed the precision marketing data attribute deriving from the e-commerce platform based on a Generative Adversarial Network (GAN). The authors also optimized the association rules for achieving accurate data mining. Experimental results of the proposed solution showed that the integrity of the data mining was steadily at 80%, while the relative and average error were lower than 0.3% [93].

Additionally, Automatic Speech Recognition (ASR) systems used in the e-commerce environments assist in many tasks necessary for the workflow in such ecosystems, such as browsing catalogs, shopping, ordering food, or scheduling deliveries. Shenoy et al. used a hybrid ASR system in order to adapt a Transformer XL Normalized Maximum Likelihood (NML) for speech conversations on e-commerce. With the use of semantic embeddings, the authors proved that the domain general TXL NML for the e-commerce task of re-scoring can be effectively implemented, while they introduced a TXL loss function used for training in order to predict content words together with language modeling task, which, when combined with BERT fusion, has better overall performance on ASR and Natural Language Understanding (NLU) metrics. In addition to ASR, e-commerce, as a business activity in nature, relies on the fusion of information, cost, and efficiency advantages of tourism while it has also played an important role in competitiveness in the tourism industry. E-commerce has assisted in the way the tourism services have promoted their level, quality, as well as the healthy and rapid development. In that light, Wei et al. proposed a multi-data fusion ANN model to predict tourism data for them to analyze and research. The authors also explained the influence of e-commerce on the tourism factors of production, such as optimizing those factors, improving the competitiveness, and optimizing industrial structure. The authors researched the fundamental reason that e-commerce can upgrade the tourism industry in China in order to find a way to have better promotion of China’s tourism [94].

Finally, fusion techniques are also utilized in methodologies for click conversion rate evaluation. Song et al. presented a methodology for advertising click rate evaluation in e-commerce. In this paper, a big data information sampling model was initially utilized for mining click rate evaluation information. In the context of the click conversion rate evaluation, adaptive mining and fusion clustering processing, statistical feature analysis, and fuzzy feature clustering as well as fuzzy genetic optimization methods were used. Testing of the proposed methodology showcased high accuracy of evaluation and prediction as well as good convergence [95].

### 4.4. Customer-Related

#### 4.4.1. Purchase Behavior Prediction

The shopping behavior of customers in e-commerce platforms is characterized by a high degree of granularity and data sparsity and the prediction of this behavior is a rather challenging task. Xu et al. proposed a purchase behavior prediction model for e-commerce platform users, based on information fusion and ensemble learning. Four base learners of different categories were selected, and the meta-learners utilized the stable LR algorithms in order to get the final information fusion and ensemble learning stacking model [96]. The methodology was tested on a dataset that is publicly available, yielding a high F1-score of 98.4% in purchase prediction, which was about 0.09% higher than the optimal base models. The training speed was also very satisfactory. In the same direction, Hu et al. described a fusion algorithm for predicting the purchase behavior, which was based on LR and SVM algorithms [97]. The predicting performance of the proposed hybrid model was evaluated on a dataset deriving from a real-world e-commerce platform and was found to provide better results as compared to the LR and SVM methodologies, being implemented individually. A similar fusion algorithm was also proposed by Liu et al. for predicting customer repeated purchase intention. In this research work, authors first analyzed customer behavior data in order to obtain rules related to purchase behavior and then utilized a fusion model based on a linear LR and XGBoost. This fusion model was found to outperform the individual models, also helping to effectively filter the utilized features [98]. Finally, Wang et al., based on points of interest (POIs), managed to predict the behavior sequence trajectory, which is a very important step in smart city realization as the IoT and social media are emerging, by proposing a trust-enhanced collaborative filtering framework based on POI recommendation. A fusion model was also applied by performing the network learning technique on a user-covisiting network by also taking into consideration the geographic influence and temporal influence of POIs [99].

#### 4.4.2. Satisfaction Prediction

Customer satisfaction is also a very important aspect when talking about sustainability and profitability of a company’s e-commerce or otherwise. So, it is very important within such an environment to be able to measure the satisfaction of the customers in order to achieve the best possible results for the company. In that light, Kumar et al. described a fusion approach where features deriving from EEG (electroencephalogram) signals were combined with sentiment analysis data websites for achieving rating prediction for consumer products [100]. After the feature extraction from ECG, customer reviews were crawled from e-commerce websites and sentiment analysis was performed. Soon after that, an RF regression technique was applied on reviews in order to predict the rating of unknown products. In addition to this, an ABC optimization approach was used to increase the framework performance by fusing local and global ratings. The experimental results indicated decreased RMSE in ratings as compared to the unimodal schemes. Sentiment analysis was also used in the work performed by Ajitha et al. where they proposed a system using a lexicon-based model, which was able to classify the feelings of the customers based on their feedback taken from emails, tweets, call center, and surveys. The authors used a Machine Learning-based fusion technique that categorized the feedback as positive, negative, or neutral [101]. On the other hand, the methodology proposed by Abbasimehr and Shabani, used a time series forecasting component [102] that fuses the results of linear and non-linear models. This method provided the advantage of using the known information about the performance of the techniques in the forecasting, which was then used by the fusion component in order to assign the proper weights to the components included in the methodology, being the clustering and the forecasting. After following the steps of clustering through time series clustering and proceeding to forecasting by predicting each segment by time series forecasting, the goal of predicting the future behavior was achieved.

### 4.5. Summary of the Analyzed Solutions

The main categories, subcategories as well as the respective citations for the solutions analyzed in this literature review can be found in Table 2.

**Table 2:** Applications of fusion techniques in e-commerce environments.

Category	Sub-Category	Publication	Number of Publications per Subcategory
Product-related	Product Classification/Description	[31,32,33,34,40]	5
	Customs Classification	[41,42]	2
	Goods Information Inspection	[43,44]	2
	Goods Demand Forecasting	[45,46,47]	3
	Shipping and Route Optimization	[48,49,50,51,52,53]	6
	Supply Chain Management	[55,56,57,58,59]	5
Economic-related	Financial and Credit Risk Prediction	[60,61,62]	3
	Price Prediction	[63,64,65]	3
	Financial and Credit Fraud detection	[66,67,68,69,70,71]	6
Business-related	Business Intelligence and Decision support	[73,74,75,76,77,78,79]	7
	Information Quality Assessment	[4,80,81]	3
	Recommendation Systems	[82,83,84,85,86,87,88]	7
	Marketing Optimization	[90,91,92,93,94,95]	6
Customer-related	Purchase Behavior Prediction	[96,97,98,99]	4
	Satisfaction Prediction	[100,101,102]	3
Total			65

## 5. Discussion and Conclusions

The present paper surveyed a wide range of fusion-related applications in e-commerce environments. A total of 65 different publications were analyzed, and a 4-fold categorization was introduced for the e-commerce-related challenges addressed by the analyzed papers. The categorization includes product-related, economic-related, business-related, and customer related applications, and 15 subcategories were also introduced. Many of the categories and subcategories are strongly interrelated, and as such the categorization should not be considered as strict and exhaustive, but rather as a means to provide the reader with a clearer understanding of the challenges addressed by each application. The steps the authors followed for this paper and the sources they utilized were described in detail.

The product-related applications were highly diverse, addressing many different aspects and challenges related to products (e.g., shipping, product classification, product description, goods demand forecasting, shipping), while other solutions integrated the whole supply chain. Aspects regarding cross-border e-commerce services as well as environmental sustainability were also taken into account by certain applications.

On the other hand, the majority of the economic-related solutions mainly focused on methodologies for predicting and mitigating financial fraud or financial risk, which also seems to be under great consideration for many of the authors due to the fact that many different machine learning techniques were proposed for that purpose.

As far as the business-related applications are concerned, there is a very large number of publications regarding fusion-based frameworks and solutions that mainly focus on supporting decision making of business stakeholders. In the context of these solutions, various sources are utilized (e.g., user reviews, social media, user characteristics, environmental factors, financial factors). Fusion techniques, also come in handy in several marketing optimization solutions.

Finally, regarding the customer-related solutions, which are very important for e-commerce companies’ profitability, they have mostly focused on the optimization of already tested models through fusion. Most common algorithms used are those for sentiment analysis as well as forecasting machine learning techniques.

Overall, the results showed that many of the papers cited focused on already applied solutions to a specific problem and tried to update and optimize those solutions by combining them through model fusion or by fusing their results. In many cases included in this literature survey, the evaluation results of fused models in terms of several metrics (e.g., accuracy, precision, f1-score, recall) were better as compared to the results acquired by the models implemented in isolation. In addition, there seems to be a need for different machine learning approaches focusing on the same problem, since there is a great variety and heterogeneity in the machine learning techniques and algorithms applied, even by the same authors. Some indicative examples of models and algorithms applied in the analyzed solutions include, kNN, Kalman Filters, BERT, CNNs, LSTM, RF, XGBoost, ANNs, SVM, fuzzy logic, DTs, LR, ABC, NB, etc.

As future research directions, we propose similar literature review approaches based on some or all of the aspects included in the categorization showcased by the present paper. Other research works may use the same main categorization, followed by a subcategorization based on the specific Machine Learning methodologies applied in the context of the respective fusion applications. Except for the methodologies presented in the current paper, future research work may include additional computational intelligence algorithms, used to solve e-commerce-related problems. Indicative examples of such methods include the Monarch Butterfly Optimization (MBO) [103,104], the Earthworm Optimization Algorithm (EWA) [105], the Elephant Herding Optimization (EHO) [106], the Moth Search (MS) algorithm [107], the Slime Mould algorithm (SMA) [108], the Hunger Games Search (HGS) [109], the Runge Kutta optimizer (RUN) [110], the Colony predation algorithm (CPA) [111], and the Harris Hawks optimization (HHO) [112].

## Figures and Tables

**Table 1:** Categorization of data fusion techniques based on classification criteria found in the literature.

Classification Criterion	Categories of Data Fusion Techniques	Source
Input and Output Types	Data In—Data Out (DAI, DAO), Data In—Feature Out (DAI-FEO), Feature In—Feature Out (FEI-FEO), Feature In—Decision Out (FEI-DEO), Decision In—Decision Out (DEI-DEO)	Dasarathy [9]
Abstraction Level	Signal level, Pixel level, Feature level, Decision level	Luo et al. [10]
Processing Levels	Level 0—Source Preprocessing, Level 1—Object Refinement, Level 2—Situation Assessment, Level 3—Impact Assessment, Level 4—Process Refinement	White [2]
Relation between the Input Data Sources	Complementary, Redundant, Cooperative	Durrant-Whyte [11]
Architecture Type	Centralized, Decentralized, Distributed	Castanedo [12]
